# Suboptimal multisensory processing in pediatric migraine without aura: a comparative, cross-sectional study

**DOI:** 10.1038/s41598-023-46088-x

**Published:** 2023-11-08

**Authors:** Gábor Braunitzer, Kálmán Tót, Gabriella Eördegh, András Hegedűs, Ádám Kiss, Jenő Kóbor, Ákos Pertich, Attila Nagy

**Affiliations:** 1 Laboratory for Perception and Cognition and Clinical Neuroscience, Nyírő Gyula Hospital, Lehel Utca 59-61, Budapest, 1135 Hungary; 2 Department of Physiology, Medical School, University of Szeged, Szeged, Hungary; 3 Faculty of Health Sciences and Social Studies, University of Szeged, Szeged, Hungary; 4 Department of Pediatrics and Pediatric Health Center, Medical School, University of Szeged, Szeged, Hungary

**Keywords:** Neuroscience, Psychology, Neurology

## Abstract

Alterations of sensory processing in migraine are well known. There is some evidence to suggest that multisensory processing is altered in migraine as well, but the area is underexplored, especially regarding pediatric migraine. A visual and an audiovisual version of the Rutgers Acquired Equivalence Test paradigm was administered to pediatric patients with migraine without aura (aged 7–17.5 years) and to age- and sex-matched controls. The application of audiovisual stimuli significantly facilitated associative pair learning in migraine-free children and adolescents, but not in pediatric migraine patients. The results of this study corroborate the hypothesis that multisensory processing is altered in pediatric migraine without aura.

## Introduction

It is increasingly recognized that altered sensory processing can be a core cognitive feature in migraine, both during the attacks and interictally^1^. This is especially well documented regarding visual processing. Our research group has demonstrated alterations of interictal visual processing in migraine, in both adult and pediatric patient populations, from elementary visual functions, such as contrast sensitivity, to complex tasks like visually guided associative learning^2–6^. To study visually guided associative learning, we used an adapted version of the Rutgers Acquired Equivalence Test (RAET or “fish-face” test)^6,7^ and concluded that children living with migraine without aura do not exhibit the same cognitive deficits in RAET as their adult counterparts^5^. This observation led to the hypothesis that the deficit of visual associative learning is not an inherent cognitive alteration in migraine, rather, it stems from the disease interfering with development.

The idea that migraine might be “a disorder of multisensory integration”, as Todd Schwedt put it in the first review on the topic in 2013^8^, has been around for some time, especially because of the various sensory phobias observed in migraine and the ability of sensory stimuli to trigger attacks. While Schwedt concentrated primarily on pain and the other senses in migraine, O’Hare, in the latest review on the topic, took a wider perspective and summarized the available knowledge about visual-auditory and visual-vestibular integration, too^9^. Her conclusion supports the idea that multisensory integration is altered in migraine, and she also remarks that the area is underexplored. Indeed, only a few studies are available that directly addressed this question^10–13^. Most of these studies confirmed altered multisensory processing in migraine. Only the study by Jonas and co-workers^13^ suggests otherwise, where “no general link between migraine and synesthesia nor between migraine with aura and grapheme-color synesthesia” was found. It is of interest for the present study that Di Marco and co-workers found some alterations in the sound-induced flash illusion in a pediatric migraine population^11^. At the moment, the study of the Di Marco group is the only available study on multisensory processing in pediatric migraine. In summary, most of the available evidence supports altered multisensory processing in migraine, but the question is still underexplored, especially regarding pediatric migraine.

Acquired equivalence is a specific phenomenon within the broader framework of associative learning. It involves the ability to generalize learned associations between stimuli, even when the stimuli are dissimilar in appearance^14^. In other words, acquired equivalence demonstrates how individuals or organisms can apply their learned associations to different, superficially unrelated stimuli, based on their shared outcomes or responses. This phenomenon highlights the flexibility and generalization capabilities of associative learning processes, showcasing the brain's ability to recognize similarities in learned experiences and apply them in diverse contexts.Based on the visual associative learning and acquired equivalence test we used in our previous studies on pediatric and adult migraine (RAET), we developed a multisensory (audiovisual) test (the “sound-face” test, SFT). This test is built on the same principles as RAET, but it uses sounds as antecedent stimuli instead of cartoon faces, so test subjects build pairs of auditory and visual stimuli. We have applied this new test to various populations: adults and children, healthy and diseased^15,16^. Of note, we have recently published a study where we administered RAET and SFT to healthy children and adolescents (5 to 17 years of age) and found significant multisensory gain^17^.

In this study, we sought to contribute to the underexplored area of multisensory processing in pediatric migraine. We examined children and adolescents suffering from migraine without aura and age-, sex- and intelligence-matched controls. Both patients and controls were administered two tests: RAET and SFT. Based on our knowledge from our previous studies, we hypothesized that in RAET, patients would perform at approximately the same level as controls. Regarding SFT, we set up two hypotheses: as for the control group, we hypothesized that multisensory stimuli would enhance the effectiveness of equivalence learning as before^17^. As for the migraine group, we could only rely on the findings of Di Marco and colleagues^11^ and hypothesized that the patient group would exhibit altered performance in this test.

Preliminary results from this study were presented at the Sixth Hungarian Neuroscience Meeting for Undergraduate Students, Graduate Students and Young Post-Docs (HuNDoC)—January 31, 2023.

## Results

### The studied populations

A total of 60 children and adolescents aged 7 to 17.5 years participated in the study. The patient group consisted of 30 participants and the control group involved 30 healthy subjects matched by age, sex, and level of intelligence as indicated by PM/PMC.

The mean age in the patient group was 13.75 ± 3.13 years, and the female-to-male ratio was 18 to 12. As for the control group, the mean age was 13.73 ± 3.17 years, and the female-to-male ratio was the same as in the patient group.

All participants in both the patient and the control groups were of Caucasian descent.

No participant in either group had to be excluded from the analysis for failure to complete the tasks involved in the study.

### Performance in the acquisition phase (NAT, ALER)

In the visually guided test, the median NAT was 58 (range 42–255; N = 30) in the patient group and 63.5 (range 44–119; N = 30) in the control group. The difference between the groups was not significant (U = 359, *P* = 0.181).

In the audiovisual test, the median NAT was 56 (range 41–97; N = 30) in the patient group and 51.5 (range 41–76; N = 30) in the control group. The difference was not significant in this test either (U = 358, *P* = 0.176).

In the patient group, NAT did not differ significantly between the tests (Z = 1.081, *P* = 0.280). However, in the control group, the difference was significant (Z = 3.106, *P* = 0.002, Effect size: 0.70, 1-β: 0.99).

In the visually guided test, the median ALER was 0.079 (range 0–0.294; N = 30) in the patient group and 0.083 (range 0–0.205; N = 30) in the control group. The difference was not significant (U = 390, *P* = 0.378).

In the audiovisual test, the median ALER was 0.057 (range 0–0.144; N = 30) in the patient group and 0.039 (range 0–0.139; N = 30) in the control group. The difference was not significant (U = 389.5, *P* = 0.375).

In the patient group, ALER did not differ significantly between the tests (Z = 1.395, *P* = 0.163). However, in the control group, ALER was significantly lower in the audiovisual test (Z = 2.725, *P* = 0.006, *d* = 0.65, 1-β: 0.99).

### Performance in the test phase (RER, GER)

In the visually guided test, the median RER was 0.042 (range 0–0.361; N = 30) in the patient group and 0.056 (range 0–0.250; N = 30) in the control group. The difference was not significantly different (U = 440, *P* = 0.886).

In the audiovisual test, the median RER was 0.028 (range 0–0.611; N = 30) in the patient group and 0.0 (range 0–0.194; N = 30) in the control group. The difference was not significant (U = 366.5, *P* = 0.197).

RER did not differ significantly between the tests in either the patient group (Z = 0.214, *P* = 0.830) or the control group, but in the control group, the difference was nearly significant (Z = 1.800, *P* = 0.072).

In the visually guided test, the median GER was 0.083 (range 0–1; N = 30) in the patient group and 0.125 (range 0–1; N = 30) in the control group. The difference was not significant (U = 426.5, *P* = 0.728).

In the audiovisual test, the median GER was 0.125 (range 0–0.667; N = 30) in the patient group and 0.042 (0–0.750; N = 30) in control group. The difference was not significant (U = 395.5, *P* = 0.401).

GER did not differ significantly between the tests either in the patient group (Z = 0.635, *P* = 0.525) or the control group (Z = 1.345, *P* = 0.179).

The performance results are summarized in Table 1.

**Table 1:** Descriptive statistics of the subjects’ behavioral performance according to the studied parameters in the visual (RAET) and the audiovisual (SFT) tests.

	NAT		Sig	ALER		Sig	RER		Sig	GER		Sig
	RAET	SFT		RAET	SFT		RAET	SFT		RAET	SFT	
PATIENT N = 30	58 (42–255)	56 (41–97)	0.280	0.079 (0–0.294)	0.057 (0–0.144)	0.163	0.042 (0–0.361)	0.028 (0–0.611)	0.830	0.083 (0–1)	0.125 (0–0.667)	0.525
CONTROL N = 30	63.5 (44–119)	51.5 (41–76)	0.002*	0.083 (0–0.205)	0.039 (0–0.139)	0.006*	0.056 (0–0.250)	0.0 (0–0.194)	0.072	0.125 (0–1)	0.042 (0–0.750)	0.179
Sig	0.181	0.176		0.378	0.375		0.886	0.197		0.728	0.401	

Sig.: significance level (p). In the significance columns, the between-tests *p* values are shown within for the given group (patient or control), and the significance row at the bottom shows the between-groups *p* values for the given test. Asterisk (*) indicates a significant difference.

### Reaction times

Reaction times did not differ significantly in any of the between-groups or between-tests comparisons, but the difference between RAET and SFT was exactly on the border of statistical significance. The results are summarized in Table 2.

**Table 2:** Reaction times in milliseconds according to the studied parameters in the visual (RAET) and the audiovisual (SFT) tests. The values are given as median (range).

	ALER		Sig	RER		Sig	GER		Sig
	RAET	SFT		RAET	SFT		RAET	SFT	
Patient N = 30	1580.9 (1081.2–2617.2)	1523.7 (979.5–3162.4)	0.845	1793.7 (1173.7–3347.7)	1612.2 (1171.0–3132.4)	0.086	2321.2 (1120.4–7209.8)	1808.6 (1187.3–3841.5)	0.050
Control N = 30	1545.1 (900.1–2646.5)	1525.4 (1075.9–2528.0)	0.910	1640.0 (1039.7–3670.2)	1491.3 (1081.3–2492.3)	0.060	2073.1 (1137.4–5018.8)	1853.3 (1209.2–3784.8)	0.117
Sig	0.404	0.673		0.290	0.297		0.582	0.865	

NAT is not shown as reaction time is not applicable to this parameter. Sig.: significance level (p). In the significance columns, the between-tests *p* values are shown for the given group (patient or control), and the significance row at the bottom shows the between-groups *p* values for the given test.

## Discussion

The findings of this study are simple and easy to summarize: compared to the application of purely visual antecedents and consequents, audiovisual stimuli significantly facilitated associative pair learning in migraine-free children and adolescents, but not in pediatric migraine patients. Apart from this difference, both groups performed equally well in all the other phases of the applied paradigm, regardless of whether the stimuli were visual or audiovisual. These findings confirmed our hypotheses and support the idea that multisensory processing is affected in migraine.

The latter observation was, at least partly, expected. Earlier we demonstrated with RAET that pediatric migraine patients do not perform worse in this paradigm than age-, sex-, and intelligence-matched controls^5^, so the findings regarding visual equivalence learning corroborate our previous results. The fact that the same is true for SFT suggests that the two tests share essentially the same underlying neural network^18^, regardless of stimulus modality, which is logical as the paradigm is the same, only the stimuli differ. Interestingly, enhanced performance (i.e. more efficient learning) was not accompanied by significantly shorter reaction times, as one would expect regarding multisensory facilitation^19^. In addition, the same effect is not seen in healthy adults^15^, so this is most probably not a classical case of multisensory facilitation. Instead, we suggest, that this is a relative enhancement in a period when the neural network behind visual associative pair learning is not yet fully mature^20,21^. That is, migraine-free children and adolescents can make use of the multimodality of the stimuli to enhance the performance of a system that is still in development. The question is why children and adolescents with migraine without aura cannot do so.

The neural background of the paradigm of RAET and SFT is well established. It has been demonstrated that the acquisition phase (feedback-supported pair learning) depends on the substantia nigra- striatum loop^7,18,22^. In addition, the basal ganglia comprise a multisensory convergence zone^23^. Indeed, there is evidence to suggest that migraine damages the basal ganglia^24,25^. However, such observations were made in adult patients (middle-aged and beyond, sometimes characterized by a high attack frequency), which suggests that the damage is caused by repeated exposure to the attacks for a longer period. Maleki and colleagues explicitly state that the damage they observed was proportional to attack frequency^24^, and several other studies came to the same conclusion in connection with microstructural brain damage in migraine without aura^25–27^. Unfortunately, we have no accurate data regarding our patients’ attack patterns prior to the diagnosis. This is obviously a limitation, but one that we cannot efficiently address. Parents start to carefully observe their children’s headache once the diagnosis has been set up (or if they cannot provide enough information for the diagnosis and are told to keep a record for a given period). As for the time before the diagnosis, they can sometimes tell us much about the headache, but not with the accuracy that analysis would require. Still, an outstandingly high attack frequency is something that a parent would report, and we had no such reports. Finally, suboptimal basal ganglia function due to damage should have shown also in RAET^7^, but the patients learned visual stimulus pairs just as successfully as the controls. All in all, even if a decisive answer could be expected only from neuroimaging, we consider it highly unlikely that our observation could be explained with attack-related microstructural damage in these young patients.

The basal ganglia accomplish their tasks as part of cortico-subcortical loops, and they have rich cortical connectivity^28^. Altered excitability of the cortex in migraine with or without aura is an often- reported phenomenon, and it appears to be an inherent feature of the migraine brain. Most studies report hyperexcitability, especially but not exclusively of the visual cortices^8,10,11,29,30^, while some authors argue that the cortex is hypoexcitable between the attacks. Di Marco and colleagues argued that the alterations they observed in the sound-induced flash illusion in a pediatric migraine population (without aura) could be put down to cortical hyperexcitability^11^. In a previous EEG study, in which we investigated RAET and SFT, we demonstrated that multimodal cues require less prominent, but more synchronized cortical contribution^31^. It is easy to see that aberrant cortical excitability in various cortical areas does not promote synchronized activation and it can also interfere with the communication and cooperation between the cortex and the still developing basal ganglia. We hypothesize that such miscommunication could lead to the observed phenomenon, especially if the contribution of the cortex is necessary for the gain seen in the healthy controls. This, however, is only a rudimentary hypothesis formulated in an almost complete lack of published data on multisensory processes in childhood migraine.

The limitations of this study include the relatively low number of participants (due to and partially offset by the strict application of the diagnostic criteria), that we did not have attack frequency data from the period before the diagnosis (see discussion above), and that we did not differentiate between children and adolescents. This latter could be problematic in the case of a larger sample, as it would clearly mean missing the potential effects that adolescence exerts on the developing human brain^32^. Indeed, we could have defined an arbitrary age limit to conduct sub-analyses, based, for instance, on statistical data, but the small sample size of our study would still question the validity of such sub-analyses. In addition, defining the onset of adolescence poses a challenge due to the lack of objective indicators and the variability in puberty onset, which is not only individual-specific but also varies between sexes. It would be intriguing to compare children with adolescents by all means, but the mentioned issues could be counterbalanced only by a much larger sample size.

In our opinion, however, these limitations do not interfere with the validity of the observation that audiovisual stimuli significantly facilitate associative pair learning in migraine-free children and adolescents, but not in pediatric migraine without aura.

The results of this study corroborate the hypothesis that multisensory processing is altered in pediatric migraine without aura.

## Methods

Please note that some parts in this section may overlap with our previous work^5,15–17,20,31^. This is because we regularly apply the described cognitive test paradigm (in both is visual and audiovisual form) in different populations, always in the same format and under the same circumstances.

### Participants

The patients were recruited from the Department of Pediatrics, Faculty of Medicine, University of Szeged, Hungary. The study period lasted from January 2020 through October 2020. Inclusion criteria were a new diagnosis of migraine without aura as determined by the same pediatric neurologist (JK) according to ICHD-3 and age in the pediatric range (maximum age: 18 years). Only patients with no other neurological, psychiatric, ophthalmological, or otological disorder were eligible for this study. A negative history was verified from the patients’ files. A further exclusion criterion was color vision deficiency, which was tested using Ishihara plates before testing. For all patients, at least five days had passed since the last attack at the time of testing. For an optimal matching of controls, the patients were also tested for intelligence with the standard (over 12 years of age) or colored (under 12 years of age) version of Raven’s progressive matrices (PM/CPM). Both patients and controls were free of psychotropic medication, including any migraine treatment at the time of testing. The sample size was determined by the rigorous application of the diagnostic and inclusion/ exclusion criteria. In total, 34 newly diagnosed pediatric patients with migraine were approached during the study period. One of them was excluded for suspected comorbid epilepsy, one for signs of excessive anxiety, one for anomalous color vision, and one for suspected aura. This left us with the final sample of 30 patients.

The study was carried out according to a one case-one control design. Controls were selected from a pool of 182 healthy children and adolescents previously recruited from primary schools and secondary schools in Szeged, Hungary to build a control pool for this type of study. Members of the control group had no history of any kind of headache, and they were also free of any kind of neurological, psychiatric, ophthalmological, or otologic disorder. Like in the case of the study population, only controls with intact color vision were eligible, as assessed with the Ishihara plates, and they were also tested with PM. Matching of cases and controls was based on age (with a tolerance limit of ± 6 months), sex, and score achieved on PM/CPM.

The study protocol conformed to the ethical principles of the Declaration of Helsinki in all aspects. Before testing, participants and their guardians were informed about the background, aims, and procedures of the study both orally and in written form. None of the subjects received any compensation for their involvement, and they were informed that the study had merely scientific purposes without direct diagnostic or therapeutic use, and they were free to quit at any time. All recruitment and protocols were conducted with written informed consent and with the approval of the Regional Research Ethics Committee for Medical Research at the University of Szeged, Hungary (Reg. No. 27/2020-SZTE).

### The study setting

The test took a place in a quiet room. The tests were executed on a personal computer, with a cathode-ray tube (CRT) screen (refresh rate 60 Hz). The participants were sitting at a standard distance (114 cm) from the computer screen. The auditory stimuli were administered through Sennheiser HD439 over-ear headphones (Sennheiser, Germany) at SPL = 60 dB, to both ears at the same time. On the keyboard, the M and the X keys were labeled as “right” and “left”, respectively. The participants were tested individually, one after the other. There was no time limit or forced responses.

### The applied tests

In this study, two versions of the same test paradigm were used: RAET (visual) and SFT (audiovisual). The two main phases of both tests are the acquisition and the test phases. The test phase consists of two parts: retrieval and generalization/transfer.

In the acquisition phase, the participants had to learn the association between two stimuli (an antecedent and a consequent) through trial-and-error learning. The subject indicated his or her guess as to which consequent is associated with any given antecedent by pressing the “left” or the “right” button (see above). The computer provided immediate visual feedback on the correctness of the guess: a green checkmark for a correct guess and a red X for an incorrect guess. The subject had to achieve a certain number of consecutive correct answers after the presentation of each new association to be allowed to proceed. This number was 4 when the first association was presented, and it increased by 2 upon the presentation of each association that followed (up to a maximum of 12). Thus, the length of the acquisition phase varied among the participants, depending upon how efficiently they learned. There were altogether 4 antecedents and 4 consequents. Of the 8 possible associations, 6 were taught in the acquisition phase. During the acquisition phase, the subjects also learned implicitly that certain antecedents are equivalent in terms of their relation to the consequents (for instance, it is always the male faces that are associated with the green fish; see below). Such information is crucial for the correct identification of the new associations in the transfer part of the test phase.

In the test phase, no further feedback was given about the correctness of the responses (guesses), and participants had to recall the already acquired six associations. This is the retrieval part of the test phase. Two new, hitherto unknown associations were also presented, which were derivable from the six known associations. The task of the subjects was to correctly identify these new associations based on their previous knowledge about the antecedents. This is the generalization/transfer part of the test phase. The subjects were not informed that new associations could crop up during the test phase, only that their task was the same, but without feedback. The test phase consisted invariably of 48 trials (36 recall trials and 12 transfer trials in a random sequence).

For visual testing, an adapted version of the original RAET by Myers and co-workers^7^ was applied. The testing software was re-written for Microsoft Windows and translated into the Hungarian language with the written permission of Professor Catherine E. Myers at Rutgers University, NJ. In this test, the antecedents are cartoon faces of a woman (A1), a girl (A2), a man (B1), and a boy (B2), and the consequents are yellow (X1), red (X2), green (Y1) and blue (Y2) fish.

The audiovisual test (SFT) was developed in our laboratory, following the principles of RAET. In this test, the antecedents are sounds (cat’s meow, the sound of an engine starting, a guitar chord, and a woman saying a Hungarian word—A1, A2, B1, B2) and the consequents are visual stimuli (the four cartoon faces from the visual paradigm). In each trial, the subject heard one of the antecedents, and saw two different consequents on the right and left sides of the screen. The task was always to choose the correct consequent, as described above.

**Figure 1:**
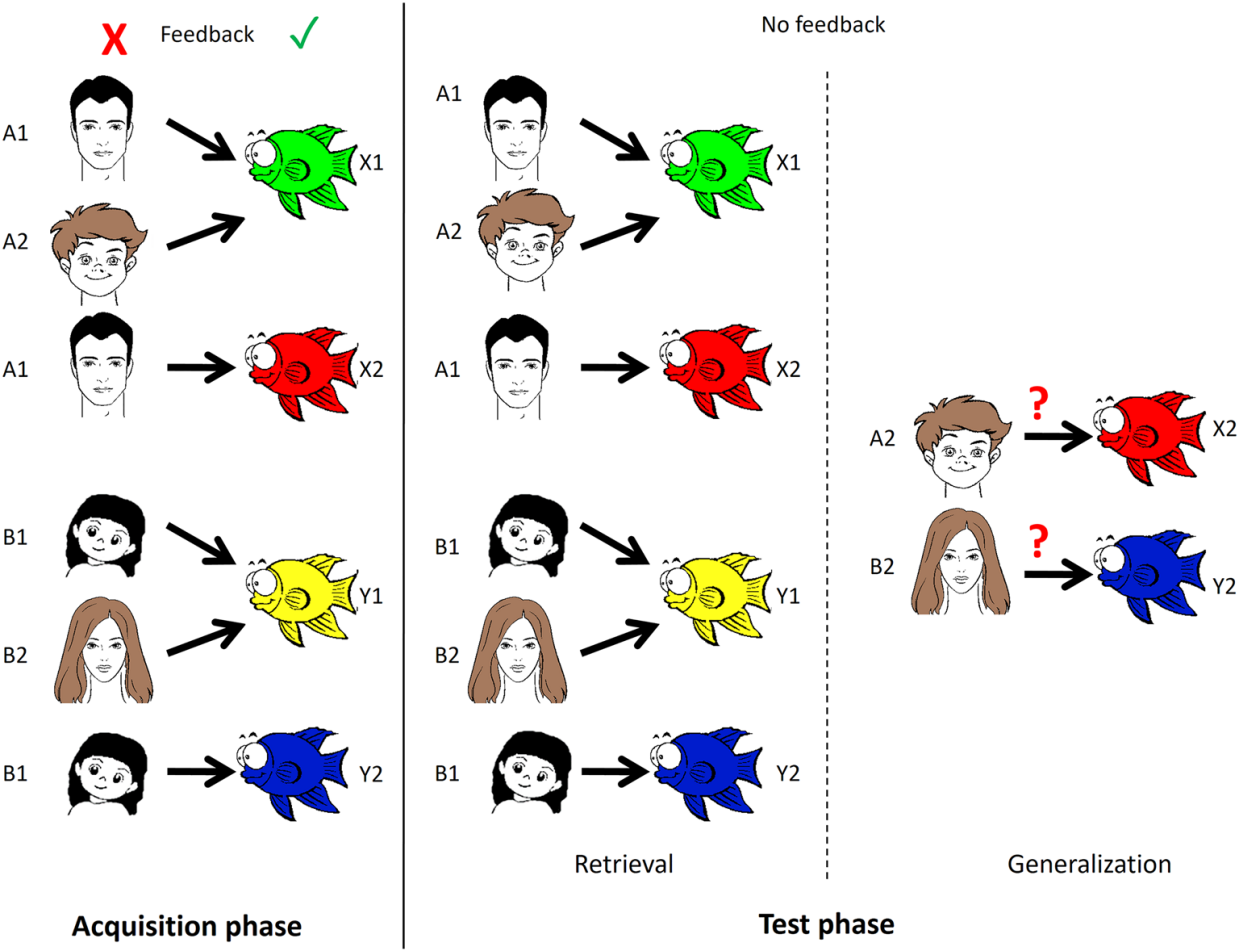
A summary of the visual test. In the acquisition phase, the task of the subject is to learn visual stimulus pairs by trial-and-error learning supported with feedback. In the test phase, the retrieval of the previously learned stimulus pairs is tested (retrieval) and new, hitherto unknown associations are also presented, which are derivable from the previously learned (known) associations (generalization). No feedback is given in the test phase.

**Figure 2:**
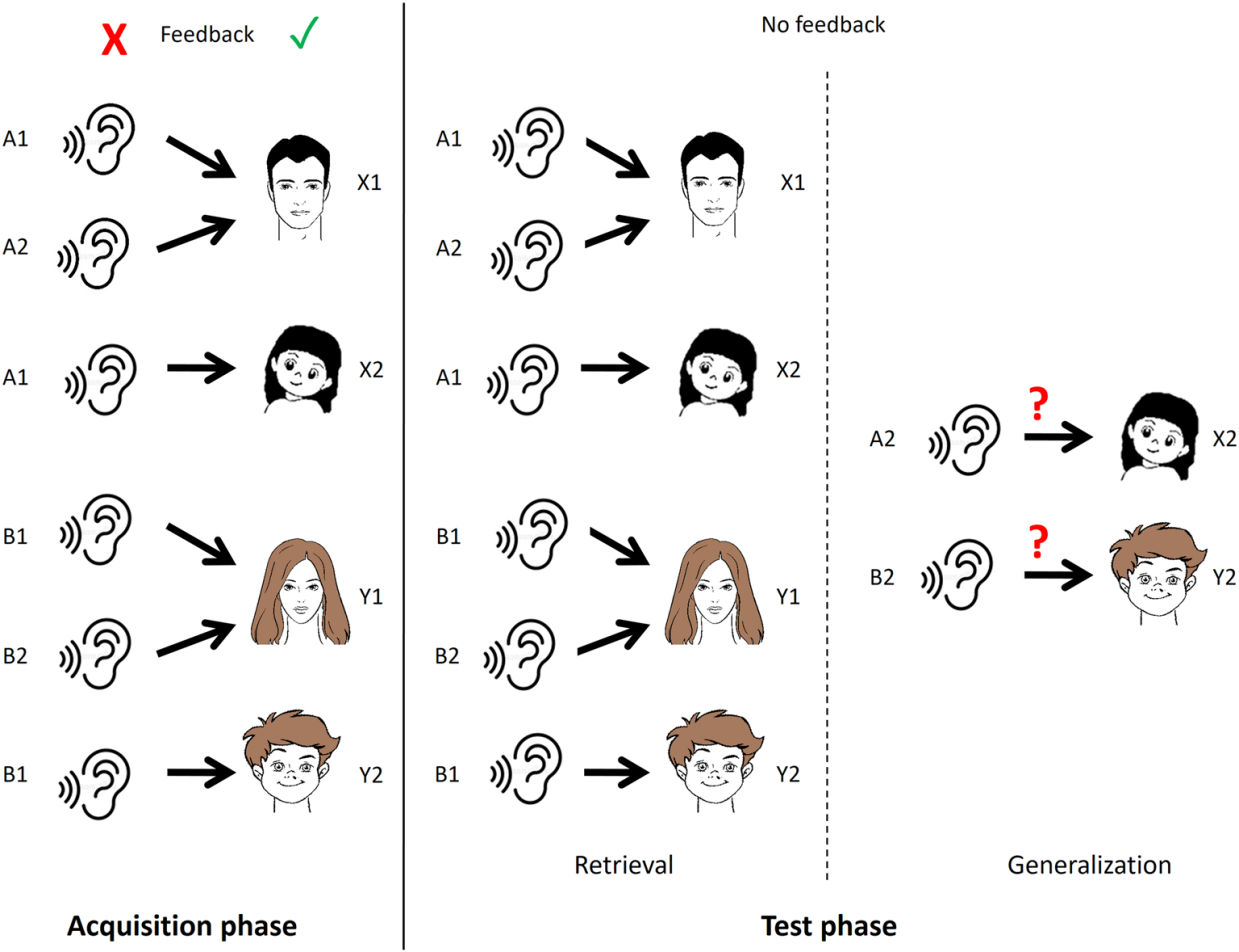
A summary of the audiovisual test. In the acquisition phase, the task of the subject is to associatively learn audiovisual stimulus pairs by trial-and-error learning supported with feedback. In the test phase, the retrieval of the previously learned stimulus pairs is tested (retrieval) and new, hitherto unknown associations are also presented, which are derivable from the previously learned (known) associations (generalization). No feedback is given in the test phase.

For a summary of RAET and SFT, see Figs. 1 and 2.

All subjects completed both tests, but the order of administration differed across the subjects to counterbalance carryover.

It is important to note that RAET was initially developed to study changes in clinical patients with structural brain damage. Therefore, its use in populations with milder deficits or healthy controls raises concerns about potential ceiling effects. In our laboratory, we have collected extensive data using this test from healthy volunteers, migraine patients (both pediatric and adult), and patients with various neurological and psychiatric conditions without structural brain damage^5,6,16,20^. We have not observed such an effect.

### Data analysis

The performance of the participants was described with four parameters: the number of trials necessary for the completion of the acquisition phase (NAT), association learning error ratio (the ratio of incorrect choices during the acquisition trials, ALER), retrieval error ratio (RER), and generalization error ratio (GER).

We also measured reaction times (RT) for acquisition, retrieval, and generalization with millisecond accuracy. Values exceeding 3 standard deviations were excluded from the analysis.

The statistical analysis was performed in Statistica 13.5.0.17 (TIBCO Software Inc., USA). According to the Shapiro–Wilk normality test, the data did not meet the criterion of normality, wherefore hypothesis testing was performed with nonparametric tests. For the between-groups (patient vs. control) comparisons within the same paradigm (visual or audiovisual), the Mann–Whitney U test was applied. For the comparisons between the paradigms within the same group, the Wilcoxon matched-pairs test was used. Effect size (Cohen’s *d*) and power calculations were performed for the significant differences in G*Power 3.1.9.4 (Universität Düsseldorf, Germany).

### Ethics approval and consent to participate

All recruitment and protocols were conducted with written informed consent and with the approval of the Regional Research Ethics Committee for Medical Research at the University of Szeged, Hungary (Reg. No. 27/2020-SZTE).

### Supplementary Information

Supplementary Information.

## Data Availability

The datasets generated and/or analyzed during the current study are available on Figshare: 10.6084/m9.figshare.20938297.v1 (Supplementary file 1).
